# Programmed Death‐Ligand 1 Expression Predicts Poor Prognosis in Patients With Early‐Stage Non‐Small‐Cell Lung Cancer Undergoing Stereotactic Body Radiotherapy

**DOI:** 10.1111/1759-7714.70296

**Published:** 2026-05-01

**Authors:** Jongmoo Park, Ji Woon Yea, Jae Won Park, Yoon Young Jo, Se An Oh, Jaehyeon Park

**Affiliations:** ^1^ Department of Radiation Oncology, School of Medicine Kyungpook National University Daegu South Korea; ^2^ Department of Radiation Oncology Yeungnam University College of Medicine Daegu South Korea

**Keywords:** non‐small‐cell lung cancer, programmed death‐ligand 1, stereotactic body radiotherapy

## Abstract

**Background:**

Stereotactic body radiotherapy (SBRT) is the standard treatment for medically inoperable early‐stage, non‐small‐cell lung cancer (NSCLC). Programmed death‐ligand 1 (PD‐L1) is a well‐known biomarker for predicting immunotherapy responses; however, its prognostic significance in early‐stage NSCLC treated with SBRT remains unclear. Here, we evaluated the prognostic significance of PD‐L1 expression in this setting.

**Methods:**

We retrospectively analyzed patients with early‐stage NSCLC who underwent SBRT. PD‐L1 expression was assessed using the SP263 immunohistochemistry assay and quantified by tumor proportion score. We evaluated the prognostic impact of PD‐L1 as a continuous variable and used an exploratory 2% cutoff derived from receiver operating characteristic analysis. Clinical outcomes, including local recurrence‐free survival (LRFS), recurrence‐free survival (RFS), disease‐free survival (DFS), and overall survival (OS), were compared. Cox proportional hazards models were used for multivariable analysis.

**Results:**

A total of 54 patients were included. SBRT achieved a 2‐year LRFS rate of 98%. As a continuous variable, higher PD‐L1 expression was independently associated with inferior RFS (hazard ratio (HR): 1.07, *p* < 0.01), DFS (HR: 1.06, *p* < 0.01), and OS (HR: 1.04, *p* < 0.01). The PD‐L1‐positive group had a higher incidence of regional recurrence (30.0% vs. 5.9%, *p* = 0.041). The exploratory 2% cutoff identified a subgroup with significantly worse survival in univariable analyses, although it did not retain independent significance in the multivariable analysis.

**Conclusions:**

PD‐L1 expression is independently associated with worse survival outcomes in patients with early‐stage NSCLC treated with SBRT, indicating its potential as a prognostic biomarker for risk stratification.

## Graphical abstract

PD‐L1 expression is independently associated with inferior survival outcomes and increased regional recurrence in early‐stage NSCLC treated with SBRT. These findings suggest that PD‐L1 status serves as a prognostic biomarker for identifying high‐risk patients who may benefit from intensified systemic strategies, such as combining SBRT with immunotherapy.

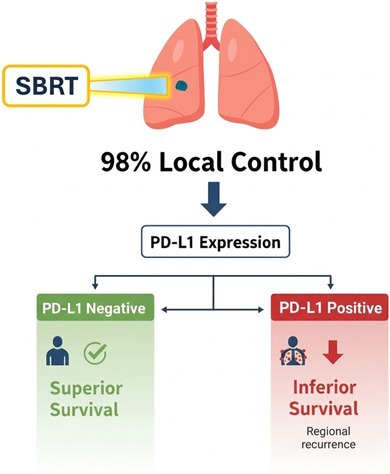

## 1 Introduction

Lung cancer is the third most common malignancy and the leading cause of cancer‐related deaths in South Korea. In 2024, there were 34 404 new cases of lung cancer and 18 278 deaths reported [1]. According to the Korean Central Cancer Registry, approximately 28% of patients with non‐small‐cell lung cancer (NSCLC) were diagnosed at stage I [2]. Surgical resection has long been considered the first‐line treatment for early‐stage NSCLC and has shown favorable clinical outcomes, with 5‐year overall survival (OS) and disease‐free survival (DFS) rates of 73.5%–88.2% and 65.5%–80.3%, respectively [3, 4]. However, for patients who are medically inoperable due to comorbidities, such as heart disease or poor pulmonary function, stereotactic body radiotherapy (SBRT) is recommended as an alternative treatment [5].

SBRT is a type of radiotherapy (RT) that delivers highly precise and intense doses of radiation to effectively eliminate cancer cells. SBRT offers better local control, toxicity, and treatment convenience [6]. It has shown low toxicity levels and similar clinical outcomes to surgery, including high local control (88%–98%) and favorable 3‐year OS rates (55%–60%) [7, 8, 9]. Nevertheless, the reported DFS of 48%–55% seems suboptimal considering the high local control rates achieved with SBRT.

Programmed death‐ligand 1 (PD‐L1) is a transmembrane protein essential for regulating the immune system by interacting with the programmed cell‐death protein 1 (PD‐1) receptor on T cells. Under normal physiological conditions, PD‐L1 helps prevent excessive immune responses and autoimmune reactions. However, in NSCLC and other malignancies, tumor cells overexpress PD‐L1, allowing them to evade immune system attacks by suppressing T cell activity [10, 11]. This mechanism has led researchers to identify PD‐L1 as a negative prognostic factor for survival in several solid tumors [12]. For instance, in NSCLC, a meta‐analysis conducted by Tuminello et al. which included patients with surgically resected NSCLC reported that higher PD‐L1 expression was associated with worse OS (hazard ratio [HR]: 1.59, confidence interval [CI]: 1.17–2.17) [13].

SBRT stimulates the innate immune system by promoting the release of tumor‐associated antigens, which activate dendritic cells and other antigen‐presenting cells through T‐cell interactions [14, 15, 16]. This immunomodulating effect may also cause tumor regression outside the irradiated field, a phenomenon known as the abscopal effect. Given the immunological impact of SBRT, it is unclear whether PD‐L1 expression is a negative prognostic factor. In this study, we aimed to evaluate whether PD‐L1 expression is a significant prognostic factor for the clinical outcomes in patients with early‐stage NSCLC who underwent SBRT.

## 2 Methods

### 2.1 Patients

This retrospective study was conducted according to the ethical principles of the Declaration of Helsinki and was approved by the Institutional Review Board of Yeungnam University Hospital (YUMC 2025‐03‐013). The requirement for informed consent was waived. In this study, we included patients with early‐stage lung cancer treated at our institution between January 2017 and December 2023. The inclusion criteria were: (1) stage I‐IIA (per the eighth edition of the American Joint Committee on Cancer [AJCC] staging system) NSCLC, (2) treatment with SBRT, (3) histologically confirmed NSCLC, and (4) pathological evaluation including PD‐L1 (SP263) testing. The exclusion criteria were: (1) prior diagnosis or treatment for another malignancy, and (2) absence of follow‐up evaluation after SBRT.

### 2.2 Stereotactic Body Radiotherapy

All patients underwent simulation using either four‐dimensional computed tomography (CT) or the “deep‐inspiration breath hold” technique to account for respiratory motion. The gross tumor volume (GTV) was delineated using contrast‐enhanced chest CT and positron emission tomography‐CT. For patients treated with respiratory gating, an internal target volume (ITV) was generated to encompass tumor motion during the gating window. The planning target volume (PTV) was defined as the GTV or ITV plus a 5–7 mm margin. The treatment plan was normalized such that the prescription dose covered at least 95% of the PTV. SBRT was delivered using 6‐MV photon beams generated by linear accelerators (Varian Medical System, Palo Alto, CA, USA). The most commonly used radiation schedule was 60 Gy/4 fx, while 60–70 Gy/8–10 fx was used for central lesions. No patient underwent adjuvant chemotherapy.

### 2.3 Data Collection

Clinicopathological characteristics, including age, sex, Eastern Cooperative Oncology Group (ECOG) performance status, smoking history, comorbidities, histology type, epidermal growth factor receptor (EGFR) status, clinical staging, and RT doses, were collected from electronic medical records. Patient identification information was temporarily accessed for case selection, and anonymized data were subsequently used for analysis. The dataset used for research purposes was accessed between March 24 and April 7, 2025. After anonymization, the authors no longer had access to any identifying information of the participants during or after data collection. PD‐L1 status was evaluated by immunohistochemistry using the Ventana SP263 assay (Roche Diagnostics Corporation, Indianapolis, IN, USA) per the manufacturer's instructions. The tumor proportion score (TPS) was defined as the percentage of viable tumor cells showing partial or complete membrane staining. The eighth edition of the AJCC and Union for International Cancer Control Tumor–Node–Metastasis classification was used for clinical staging.

After SBRT, patients underwent chest CT every 3 months for the first 2 years and every 6 months thereafter. Additionally, positron emission tomography–CT and brain magnetic resonance imaging were conducted annually to monitor distant metastasis. Tumor response was evaluated using the results from the chest CT conducted at 3–6 months after SBRT, according to the Response Evaluation Criteria in Solid Tumors. When recurrence was suspected on imaging, a multidisciplinary discussion was conducted. Based on this discussion, whenever feasible, pathological confirmation was obtained, or additional imaging modalities were used for further evaluation.

### 2.4 Statistical Analysis

Statistical analyses were conducted using IBM SPSS Statistics, version 29.0 (IBM Corp., Armonk, NY, USA). For baseline comparisons, patients were stratified into two groups based on PD‐L1 expression: positive (TPS ≥ 1%) and negative (TPS < 1%). Continuous variables were compared using the *t*‐test, while continuous variables were analyzed using Pearson's chi‐squared, Fisher's exact, or linear‐by‐linear association test as appropriate.

Survival outcomes included local recurrence‐free survival (LRFS), recurrence‐free survival (RFS), DFS, and OS. All survival intervals were measured from the start of SBRT. LRFS was defined as the time to tumor regrowth at the primary site or the same lobe. RFS included any local, regional, or distant recurrence, while DFS accounted for any recurrence or death from any cause. OS was defined as the time to death from any cause. Patients who were alive without events were censored at the last follow‐up date. Survival rates were calculated using the Kaplan–Meier method, and log‐rank tests were conducted to compare survival between the two groups.

Given the right‐skewed distribution of PD‐L1 expression (Figure S1), its prognostic impact on survival outcomes was primarily investigated as a continuous variable using Cox proportional hazards regression. HRs and 95% CI were calculated per 1% increase in PD‐L1 expression to assess the quantitative influence. For clinical interpretability and exploratory purposes, PD‐L1 expression was also evaluated using a pragmatic cutoff of 2%. Receiver operating characteristic (ROC) curve analysis was conducted to identify a mathematical threshold based on Youden's index (Table S2); however, the value was rounded to 2% to enhance practical reproducibility in clinical settings.

Cox proportional hazards models were used to investigate the impact of various factors on survival outcomes. Univariable analyses were conducted for each clinicopathologic variable. Multivariable analyses were then conducted, including variables that were statistically significant in univariable analysis with clinically relevant covariates (Charlson Comorbidity index [CCI], sex, and histology), regardless of statistical significance. The continuous and categorical forms of PLD‐L1 expression were analyzed in separate multivariable models to ensure statistical validity and avoid multicollinearity. All analyses were two‐sided, and statistical significance was defined as *p* < 0.05.

## 3 Results

**FIGURE 1:**
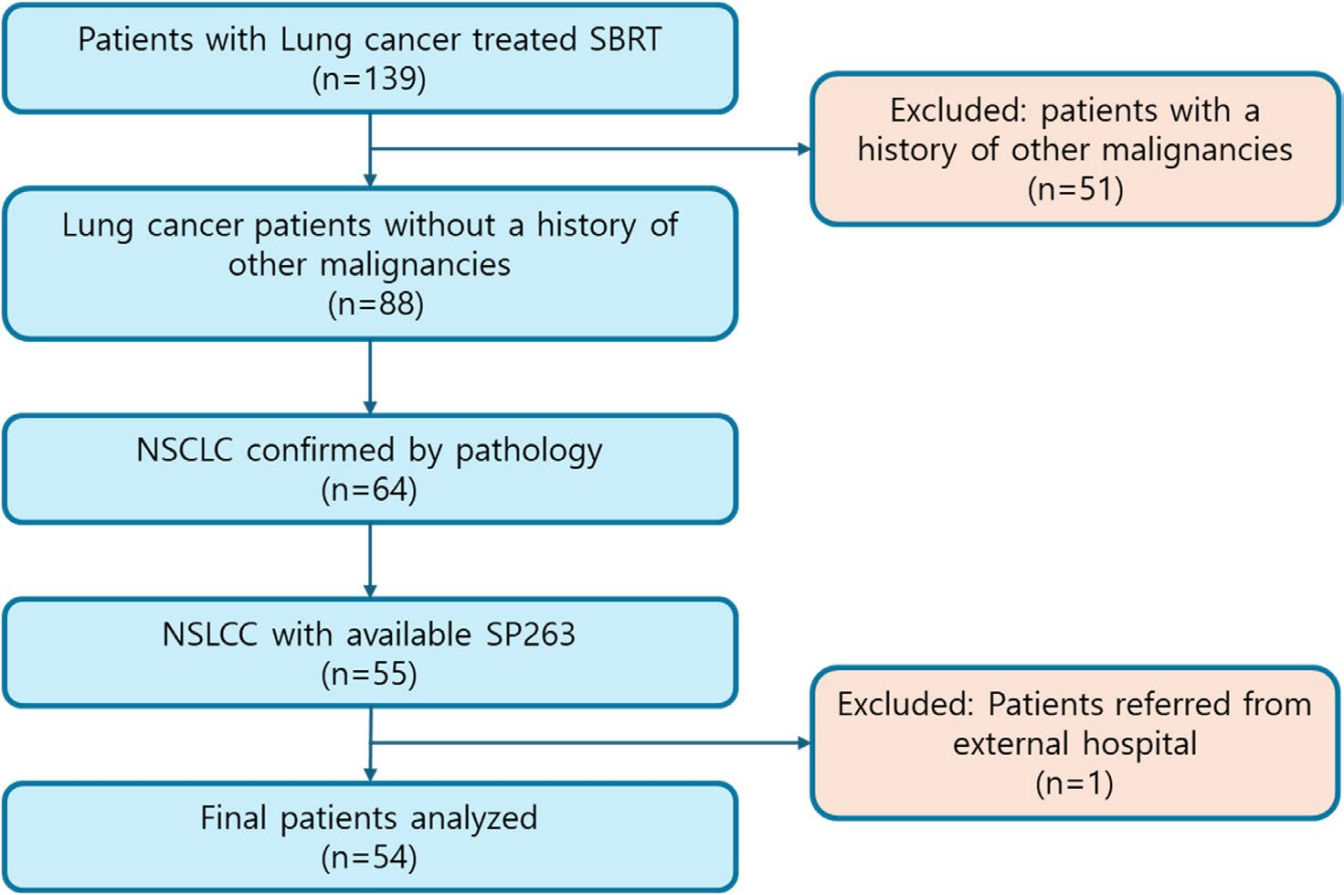
Study flowchart illustrating patient selection.

Overall, 139 patients with early‐stage lung cancer who underwent SBRT were initially identified. After excluding patients with a history of other malignancies, 88 patients remained. Subsequently, 24 patients without a pathological diagnosis and 9 who did not undergo PD‐L1 evaluation were excluded. Finally, after excluding 1 patient who did not undergo follow‐up evaluation, 54 patients were deemed eligible for this study. Figure 1 shows the patient selection process.

Overall, 54 patients were included in this study, comprising 40 (74.1%) men and 14 (25.9%) women, with a median age of 78 years (range, 65–88). Histologically, 32 patients (59.3%) had non‐squamous NSCLC, while 22 (40.7%) had squamous cell carcinoma (SCC). Based on stage classification, 17 cases (31.5%) were IA2, 20 (37.0%) were IA3, 15 (27.8%) were IB, and 2 (3.7%) were IIA. According to PD‐L1 expression, patients were categorized into two groups: PD‐L1 negative (*n* = 34) and positive (*n* = 20). Compared with the PD‐L1‐negative group, the PD‐L1‐positive group had significantly more male patients (100.0% vs. 58.8%, *p* < 0.01), a more patients with a Charlson Comorbidity Index (CCI) of ≥ 2 (82.0% vs. 41.2%, *p* < 0.01), and a higher prevalence of SCC (60.0% vs. 29.4%, *p* = 0.03). No significant differences were observed in age, ECOG performance status, smoking history, EGFR status, tumor location, clinical stage, radiation dose, or response after RT. Detailed patient characteristics are shown in Table 1.

**TABLE 1:** Patient characteristics.

	All patients (*n* = 54)	PD‐L1 negative (*n* = 34)	PD‐L1 positive (*n* = 20)	*p*
Age				0.15
Median, year (range)	78 (65–88)	79 (67–87)	77 (6–88)	
Sex				**< 0.01**
Male	40 (74.1)	20 (58.8)	20 (100.0)	
Female	14 (25.9)	14 (41.2)	0 (0.0)	
ECOG performance status				0.39
0	33 (61.1)	19 (55.9)	14 (70.0)	
1–2	21 (38.9)	15 (44.1)	6 (30.0)	
Charlson Comorbidity index				**< 0.01**
0–1	23 (42.6)	20 (58.8)	3 (15.0)	
2+	31 (57.4)	14 (41.2)	17 (82.0)	
Smoking				0.40
Never smoked	17 (31.5)	13 (38.2)	4 (20.0)	
Current	17 (31.5)	9 (26.5)	8 (40.0)	
Former	20 (37.0)	12 (35.3)	8 (40.0)	
Histology				**0.03**
Non‐squamous cell carcinoma	32 (59.3)	24 (70.6)	8 (40.0)	
Squamous cell carcinoma	22 (40.7)	10 (29.4)	12 (60.0)	
EGFR				0.10
Wild type	41 (75.9)	23 (67.6)	18 (90.0)	
Mutation	13 (24.1)	11 (32.4)	2 (10.0)	
ROS1				1.00
Wild type	45 (83.3)	28 (82.4)	17 (85.0)	
Mutation	9 (16.7)	6 (17.6)	3 (15.0)	
ALK				0.62
Wild type	50 (92.6)	32 (94.1)	18 (90.0)	
Mutation	4 (7.4)	2 (5.9)	2 (10.0)	
PD‐L1 expression				
Median, % (range)			5 (1–100)	
Tumor location				0.84
RUL	15 (27.8)	9 (26.5)	6 (30.0)	
RML	2 (3.7)	1 (2.9)	1 (5.0)	
RLL	20 (37.0)	13 (38.2)	7 (35.0)	
LUL	9 (16.7)	6 (17.6)	3 (15.0)	
LLL	8 (14.8)	5 (14.7)	3 (15.0)	
Stage				0.33
IA2	17 (31.5)	12 (35.3)	5 (25.0)	
IA3	20 (37.0)	13 (38.2)	7 (35.0)	
IB	15 (27.8)	8 (23.5)	7 (35.0)	
IIA	2 (3.7)	1 (2.9)	1 (5.0)	
Radiotherapy				0.57
Total dose	60 (39–70)	60 (39–60)	60 (50–70)	
Median, Gy (range)	4 (3–10)	4 (3–8)	4 (4–10)	
No. of fractions (range)				
Response after RT				0.38
Stable disease	18 (33.3)	15 (44.1)	3 (15.0)	
Partial response	30 (55.6)	14 (41.2)	16 (80.0)	
Complete response	6 (11.1)	5 (14.7)	1 (5.0)	

*Note:* Bold values indicate statistical significance (*p* < 0.05).

Abbreviations: ALK, anaplastic lymphoma kinase; CCI, Charlson Comorbidity Index; ECOG, Eastern Cooperative Oncology Group; EGFR, epidermal growth factor receptor; LLL, left lower lobe; LUL, left upper lobe; PD‐L1, programmed death‐ligand 1; RLL, right lower lobe; RML, right middle lobe; ROS1, c‐ros oncogene 1; RT, radiotherapy; RUL, right upper lobe.

**FIGURE 2:**
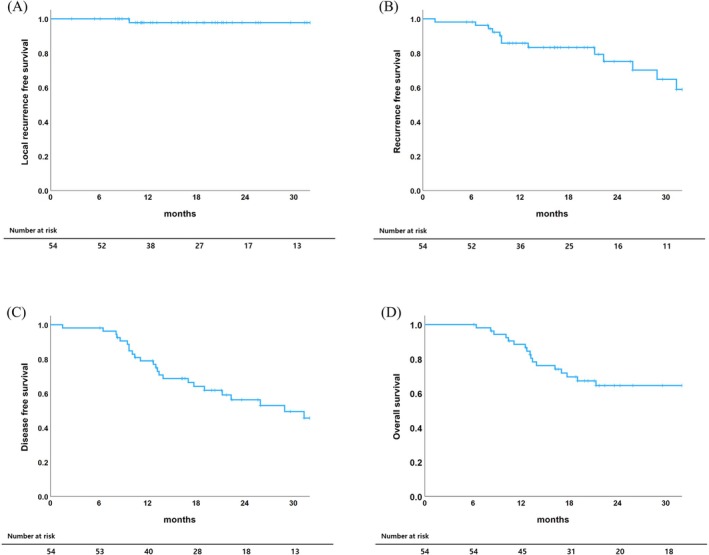
Local recurrence‐free survival (A), recurrence‐free survival (B), disease‐free survival (C), and overall survival (D) for all patients.

The median follow‐up period for all patients was 29.6 months (95% CI, 16.74–42.46), and the median OS was not reached. The 1‐ and 2‐year LRFS rates were 98% and 98%, respectively, indicating excellent local control after SBRT. Conversely, the 1‐ and 2‐year RFS rates were 86% and 75%, respectively. The 1‐ and 2‐year DFS rates were 79% and 56%, respectively. The 1‐ and 2‐year OS rates were 86% and 65%, respectively (Figure 2).

**FIGURE 3:**
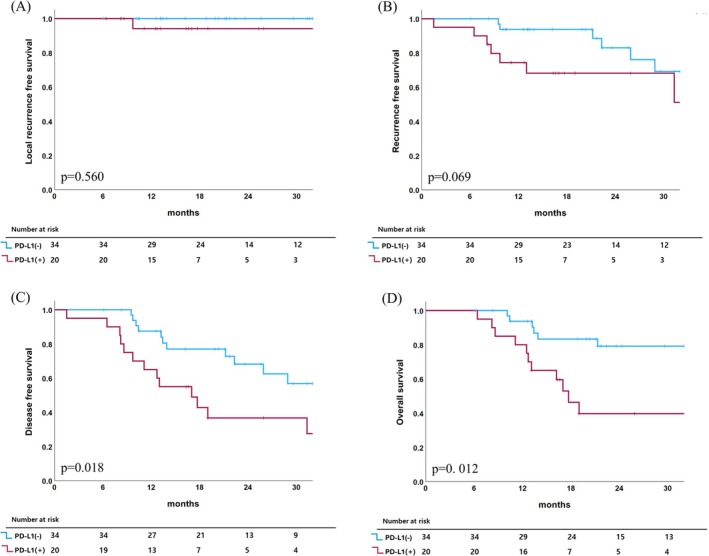
Comparison of local recurrence‐free survival (A), recurrence‐free survival (B), disease‐free survival (C), and overall survival (D) between the programmed death‐ligand 1 (PD‐L1) negative and positive groups.

Among the 54 patients, 15 (27.8%) experienced recurrence during follow‐up. While the overall incidence of recurrence between the PD‐L1‐negative and positive groups was not significantly different (20.6% vs. 40.0%, *p* = 0.124), the site‐specific failure patterns showed unique differences (Table 2). Specifically, any regional recurrence was significantly more common in the PD‐L1‐positive group than in the negative group (30.0% vs. 5.9%, *p* = 0.041). Among the eight regional recurrences, five were regional lymph node failures, with all occurring in the PD‐L1‐positive group. In contrast, no significant differences were observed in the incidence of local recurrence or distant metastasis between the two groups. Both groups showed excellent local control without a significant difference in LRFS. However, DFS and OS were significantly worse in the PD‐L1‐positive group compared to the PD‐L1‐negative group. The 1‐ and 2‐year DFS rates were 86% and 68% in the PD‐L1‐negative group and 65% and 37% in the PD‐L1‐positive group, respectively (*p* = 0.018). Similarly, the 1‐ and 2‐year OS rates were 93% and 79% in the PD‐L1‐negative group and 80% and 40% in the PD‐L1‐positive group, respectively (*p* = 0.012) (Figure 3).

**TABLE 2:** Patterns of failure between the PD‐L1‐negative group and the PD‐L1‐positive group.

	PD‐L1 negative (*n* = 34)	PD‐L1 positive (*n* = 20)	Total (*n* = 54)	*p*
Total no. of patients with recurrence	7 (20.6%)	8 (40.0%)	15 (27.8%)	0.12
Patterns of failure				
Local only	1	0	1	
Regional only	2	4	6	
Distant only	4	2	6	
Local + Regional	0	1	1	
Regional + Distant	0	1	1	
Summary by site				
Any local recurrence	1 (2.9%)	1 (5.0%)	2 (3.7%)	1.00
Any regional recurrence	2 (5.9%)	6 (30.0%)	8 (14.8%)	**0.04**
Any distant metastasis	4 (11.8%)	3 (15.0%)	7 (13.0%)	1.00

*Note:* Bold values indicate statistical significance (*p* < 0.05).

The prognostic impact of PD‐L1 was primarily evaluated as a continuous variable. In univariable analysis, PD‐L1 expression was significantly associated with worse RFS (*p* < 0.01), DFS (*p* < 0.01), and OS (*p* < 0.01). In multivariable analysis, higher PD‐L1 expression remained an independent prognostic factor for all endpoints: RFS (HR 1.07, *p* < 0.01), DFS (HR 1.06, *p* < 0.01), and OS (HR 1.04, *p* < 0.01) (Table 3). Among other clinicopathologic variables, SCC was independently associated with worse OS in multivariable analysis (HR: 3.38, 95% CI: 1.25–9.18, *p* = 0.02), while no other variables consistently retained independent prognostic significance across all survival endpoints.

**TABLE 3:** Univariate and multivariate analyses of recurrence‐free, disease‐free, and overall survival.

Clinical factor	Recurrence‐free survival				Disease‐free survival				Overall survival			
	Univariable analysis		Multivariable analysis		Univariable analysis		Multivariable analysis		Univariable analysis		Multivariable analysis	
	HR (95% CI)	*p*	HR (95% CI)	*p*	HR (95% CI)	*p*	HR (95% CI)	*p*	HR (95% CI)	*p*	HR (95% CI)	*p*
Age (years)												
≤ 78 versus > 78	0.27 (0.07–0.95)	**0.04**	0.36 (0.09–1.42)	0.05	0.58 (0.26–1.27)	0.18			1.01 (0.41–2.48)	0.98		
Sex												
Male versus female	0.45 (0.10–2.02)	0.30	0.62 (0.11–3.42)	0.58	0.24 (0.06–0.99)	**0.05**	0.73 (0.14–3.91)	0.72	0.03 (0.00–2.54)	0.12		
ECOG												
0 versus 1–2	1.02 (0.31–3.04)	0.97			1.50 (0.69–3.25)	0.30			1.77 (0.71–4.56)	0.22		
CCI												
1–0 versus 2+	1.01 (0.36–2.78)	0.99	0.32 (0.08–1.29)	0.11	1.45 (0.68–3.12)	0.34	0.64 (0.56–1.58)	0.33	2.64 (0.96–7.28)	0.06	1.44 (0.48–4.31)	0.52
Smoking												
Never smoked	1				1				1			
Former	0.64 (1.6–2.57)	0.53			1.37 (0.48–3.93)	0.55			2.66 (0.57–12.44)	0.21		
Current	1.28 (0.37–4.47)	0.70			1.69 (0.58–4.89)	0.33			3.74 (0.80–17.43)	0.09		
Histology												
Non‐SCC versus SCC	1.67 (0.60–4.67)	0.33	2.06 (0.56–7.58)	0.28	2.67 (1.25–5.68)	**0.01**	2.17 (0.88–5.37)	0.09	4.13 (1.57–10.82)	**< 0.01**	3.38 (1.25–9.18)	**0.02**
EFGR												
Wild type versus mutation	0.61 (0.17–2.17)	0.44			0.28 (0.08–0.92)	**0.04**	0.44 (0.11–1.79)	0.25	0.03 (0.00–1.61)	0.08		
ROS1												
Wild type versus mutation	1.41 (0.44–4.50)	0.56			1.68 (0.74–3.82)	0.21			2.42 (0.98–5.98)	0.06		
ALK												
Wild type versus mutation	0.58 (0.08–4.53)	0.61			0.89 (0.26–3.12)	0.86			1.30 (0.35–4.91)	0.69		
Stage												
IA2 versus IA3‐IIA	0.52 (0.19–1.45)	0.21			1.26 (0.58–2.74)	0.56			4.19 (1.23–14.30)	**0.02**		
Response after RT												
SD versus PR and CR	1.36 (0.56–4.02)	0.58			1.59 (0.72–3.54)	0.25			2.26 (0.86–7.66)	0.09		
PD‐L1												
Negative versus positive	2.45 (0.90–6.93)	0.08			2.44 (1.14–5.21)	**0.02**			3.03 (1.22–7.50)	**0.02**		
Continuous (per 1% increase)	1.06 (1.03–1.10)	**< 0.01**	1.07 (1.03–1.10)	**< 0.01**	1.05 (1.03–1.08)	**< 0.01**	1.06 (1.03–1.09)	**< 0.01**	1.04 (1.02–1.07)	**< 0.01**	1.04 (1.01–1.06)	**< 0.01**

*Note:* Bold values indicate statistical significance (*p* < 0.05).

Abbreviations: ALK, anaplastic lymphoma kinase; CCI, Charlson Comorbidity Index; CI, confidence interval; CR, complete response; ECOG, Eastern Cooperative Oncology Group; EGFR, epidermal growth factor receptor; HR, hazard ratio; PD‐L1, programmed death‐ligand 1; PR, partial response; ROS1, c‐ros oncogene 1; RT, radiotherapy; SCC, squamous cell carcinoma; SD, stable disease.

**FIGURE 4:**
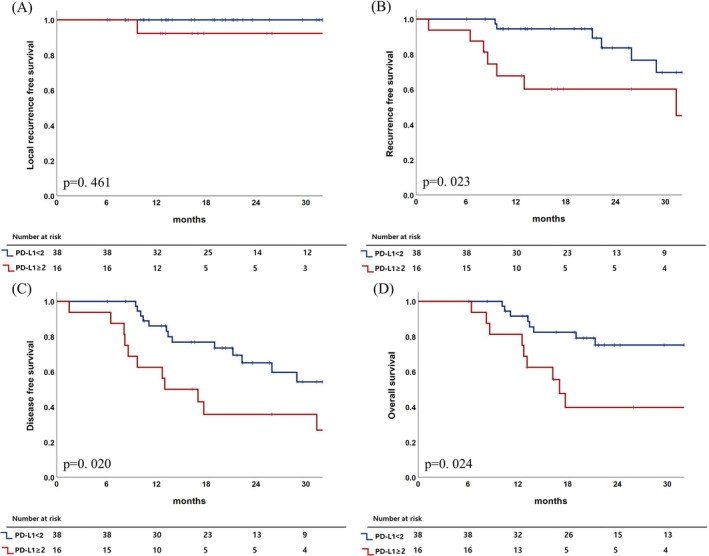
Comparison of local recurrence‐free survival (A), recurrence‐free survival (B), disease‐free survival (C), and overall survival (D) between the programmed death‐ligand 1 (PD‐L1) < 2% and PD‐L1 ≥ 2% groups.

An exploratory 2% cutoff was derived from ROC curve analysis to explore the clinical applicability of PD‐L1 expression for risk stratification (Table S1). When patients were categorized into two groups using this value, the PD‐L1 ≥ 2% group showed significantly inferior survival rates (Figure 4). The 2‐year RFS (60% vs. 84%, *p* = 0.023), DFS (36% vs. 65%, *p* = 0.020), and OS (40% vs. 75%, *p* = 0.024) were all significantly lower in the PD‐L1 ≥ 2% group than the PD‐L1 < 2% group. However, in the subsequent multivariable analysis, the 2% cutoff did not retain independent prognostic significance for RFS (*p* = 0.08), DFS (*p* = 0.11), or OS (*p* = 0.57) (Table S2).

We conducted internal validation using bootstrap resampling to assess the stability of these findings (Table S3). When analyzed as a continuous variable, PD‐L1 expression retained statistical significance with DFS and OS in multivariable bootstrap analysis. Bootstrap validation for RFS was not feasible owing to the limited number of events. For the exploratory 2% cutoff, the univariable association for RFS, DFS, and OS was reproducible in bootstrap validation. However, these associations did not show consistent stability in multivariable bootstrap analysis.

## 4 Discussion

In this study, PD‐L1 expression was independently associated with inferior RFS, DFS, and OS in patients with early‐stage NSCLC treated with SBRT. Notably, when PD‐L1 expression was modeled as a continuous variable, it consistently showed prognostic relevance across survival measures, with internal validation confirming its stability for DFS and OS. SBRT for early‐stage NSCLC achieved excellent local control, reporting a 2‐year LRFS of 98%, consistent with previous studies [7, 8, 9]. Nevertheless, the relatively low 2‐year RFS, DFS, and OS rates indicate that regional and distant metastases remain the primary patterns of failure and have a significant impact on overall prognosis. Our study revealed that this poor prognosis in the PD‐L1‐positive group was mainly due to a significantly higher rate of regional recurrence (30.0% vs. 5.9%, *p* = 0.041). To further explore the potential clinical applicability of PD‐L1 expression for risk stratification, we evaluated an exploratory 2% cutoff derived from ROC analysis. Patients with PD‐L1 ≥ 2% exhibited significantly inferior survival outcomes in univariable analyses; nevertheless, these associations were attenuated after multivariable adjustments and showed limited stability in bootstrap validation. Collectively, these findings support PD‐L1 expression as a prognostic marker in early‐stage NSCLC treated with SBRT. However, this data is insufficient to establish a definitive and clinically applicable cutoff threshold.

PD‐L1 binds to its receptor PD‐1 on activated T cells, suppressing T‐cell activation and proliferation while inhibiting cytokine secretion [10, 11]. This interaction allows tumor cells to evade immune surveillance and avoid attack by inhibiting the T‐cell‐mediated antitumor response [17]. Owing to this mechanism, PD‐L1 has also been recognized as a negative prognostic factor in other solid tumors. For instance, Xiang et al. reported that PD‐L1 expression was associated with worse 3‐year OS in various cancers, with the following HRs: esophageal cancer, 2.77 (1.78–4.30); gastric cancer, 1.63 (1.43–1.87); pancreatic cancer, 1.48 (1.06–2.06); and renal cell carcinoma, 4.14 (2.07–8.26) [12].

PD‐L1 expression has been associated with poorer prognosis in patients with resected early‐stage NSCLC. Hirai et al. reported that higher PD‐L1 expression cutoff values were associated with poor prognosis in patients with resected stage I adenocarcinoma [18]. The 5‐year OS for PDL‐1‐negative and PDL‐1‐positive patients was 85.1% versus 76.2% (cutoff, 1%, *p* = 0.249), 85.9% versus 66.7% (cutoff, 5%; *p* = 0.049), and 85.3% versus 40% (cutoff, 50%; *p* = 0.005). Sepesi et al. stratified patients with resected stage I NSCLC based on a PD‐L1 expression threshold of 4.7% and compared their clinical outcomes [19]. They found significant differences between the low and high PD‐L1 groups, with 5‐year OS rates of 94% versus 21% (*p* < 0.001) and 5‐year DFS rates of 78% versus 23% (*p* < 0.001). This study showed a similar trend in DFS, where the 2‐year DFS was 81% versus 68% (cutoff, 1%, *p* = 0.151) and 82% versus 39% (cutoff, 10%, *p* = 0.011). Furthermore, Tuminello et al. analyzed the impact of PD‐L1 expression levels on clinical outcomes in patients with resected NSCLC, using cutoff values of 1%, 5%, and 50%. They reported that high PD‐L1 expression was significantly associated with worse OS, regardless of the cutoff used (HR for 1%: 1.59 [1.17–2.17]; HR for 5%: 1.44 [1.03–2.00]; HR for 50%: 1.34 [1.04–1.73]) [13].

RT exerts immunostimulatory and immunosuppressive effects [20, 21, 22], with the dominant effect depending on the fraction size. Large fraction sizes, such as those employed in SBRT, are more immunogenic than conventional fractionation. This is because they reduce the blood volume exposed to daily radiation, increase the release of tumor‐associated antigens, promote antigen–antibody interactions, and induce tumor cell apoptosis through antitumor immune responses [16, 23, 24]. Given these immunogenic properties of SBRT, it remains uncertain whether PD‐L1 serves as a prognostic factor in patients with SBRT, as it does in patients with surgically resected NSCLC. Nevertheless, research on this topic remains limited. Short‐term outcomes were primarily evaluated in this study; nonetheless, the findings indicate that high PD‐L1 expression is associated with disease progression and poorer survival in patients undergoing SBRT, supporting its role as a negative prognostic factor.

Most recurrences after SBRT in patients with early‐stage NSCLC occur at regional and distant sites; however, adjuvant treatment is not routinely administered [25]. Ernani et al. analyzed the impact of adjuvant chemotherapy in patients with stage I–II NSCLC treated with SBRT. They found that SBRT alone resulted in significantly better survival in patients with stage I disease, with a median OS of 26.2 months compared to 21.4 months in the adjuvant chemotherapy group [26]. Conversely, in patients with stage II disease, the adjuvant chemotherapy group showed significantly improved survival (median OS: 19.9 vs. 15.0 months in the SBRT‐alone group), indicating that adjuvant chemotherapy may be beneficial for patients with larger tumors. Furthermore, Chang et al. conducted a phase II study to investigate the efficacy of adjuvant immunotherapy after SBRT [27]. Their results showed a significant improvement in the 4‐year event‐free survival, which increased from 53% to 77% among patients receiving adjuvant immunotherapy. This survival benefit was observed regardless of PD‐L1 expression and was particularly pronounced in patients who are PD‐L1‐positive. However, in contrast to these encouraging findings, the initial results of the phase III SWOG/NRG S1914 trial showed that adding immunotherapy to SBRT did not improve survival (HR: 1.15, 95% CI: 0.65–2.01, *p* = 0.63) and was associated with more grade ≥ 3 adverse events (12% in the SBRT plus immunotherapy group vs. 2% in the SBRT‐only group) [28]. Notably, in this trial, we enrolled patients with predefined high‐risk features, including tumor diameter ≥ 2 cm, standardized uptake value ≥ 6.2, and moderately to poorly differentiated histology. Based on our findings, PD‐L1 expression may also represent an important risk factor for recurrence after SBRT. Therefore, future trials should consider incorporating PD‐L1 expression into risk stratification to better identify patients who could potentially benefit from adding immunotherapy to SBRT.

In addition to PD‐L1 expression, driver mutations such as EGFR, ALK, and ROS1 are critical factors that can influence the prognosis and guide the selection of adjuvant treatments. In this study, 24.1% of the patients had an EGFR mutation; however, it did not significantly impact survival outcomes in our analysis. Nevertheless, integrating target agents with SBRT is promising. The ADAURA trial showed significant survival benefits of adjuvant tyrosine kinase inhibitor (TKI) in the surgical setting for EGFR‐mutated NSCLC (stage IB‐IIIA), where treatment with osimertinib significantly improved median DFS (65.8 vs. 28.1 months; HR 0.27, 95% CI [0.21,0.34]) and 5‐year OS (88% vs. 78%; HR 0.49, *p* < 0.001) compared to placebo [29, 30]. Furthermore, some preclinical studies have reported that TKIs can enhance radiosensitivity by blocking pro‐survival signaling and directly interfering with DNA damage responses [31, 32]. Based on these findings, assessing driver mutation status, alongside PD‐L1 expression, might be important when considering the need for adjuvant treatment in patients with early‐stage NSCLC undergoing SBRT. Further research is necessary to determine the clinical utility of biomarker‐guided strategies in this population.

This study has some limitations. First, selection bias is a significant concern as patients without histologic confirmation were excluded. This exclusion may limit the generalizability of the finding to all patients receiving SBRT. However, histologic confirmation was essential for assessing PD‐L1, and the study population represents patients for whom biomarker‐based risk stratification is clinically feasible. Second, the retrospective nature of this study may have led to an imbalance in patient distribution between the two groups. The PD‐L1‐positive group included more women, a higher CCI, and a higher prevalence of SCC. Particularly, CCI and SCC are well‐known poor prognostic factors, which may have influenced the unfavorable outcomes in the PD‐L1‐positive group. Nevertheless, multivariable analysis identified PD‐L1 as the only significant factor affecting survival across all, indicating that PD‐L1 expression could still be considered an important prognostic marker despite these imbalances. Third, this study was a single‐center analysis, with a limited number of outcome events, which may affect the stability of multivariable analysis and the generalizability of the findings despite efforts to limit model overfitting. Accordingly, validation in larger, multicenter studies is necessary. Fourth, this study only investigated short‐term outcomes owing to the short follow‐up period, which may be attributable to the older age of the patient population (median age, 78 years).

This study showed that PD‐L1 expression is significantly associated with poor prognosis in patients with early‐stage NSCLC treated with SBRT. SBRT achieved excellent local control; nonetheless, most recurrences occurred at regional and distant sites, which were significantly more common in patients with PD‐L1‐positive tumors. Notably, when PD‐L1 expression was evaluated as a continuous variable, it was an independent prognostic factor of RFS, DFS, and OS. However, an exploratory 2% cutoff identified a subgroup with inferior survival in univariable and bootstrap analysis, but it did not retain independent significance in the multivariable analysis, likely owing to the limited sample size. These findings imply that PD‐L1 expression may be a useful prognostic marker in this setting; however, further large‐scale studies are necessary to establish a standardized cutoff and explore risk‐adapted strategies combining immunotherapy with SBRT.

## Author Contributions

**Jongmoo Park:** conceptualization, methodology, data curation, validation, writing – original draft, writing – review and editing. **Ji Woon Yea:** investigation, software, resources, writing – review and editing. **Jae Won Park:** methodology, data curation, formal analysis, writing – review and editing. **Yoon Young Jo:** methodology, data curation, formal analysis, writing – review and editing. **Se An Oh:** investigation, software, resources, writing – review and editing, visualization. **Jaehyeon Park:** conceptualization, investigation, validation, writing – original draft, writing – review and editing, visualization, funding acquisition, supervision, project administration.

## Funding

This work was supported by the 2023 Yeungnam University Research Grant (223A480018).

## Conflicts of Interest

The authors declare no conflicts of interest.

## Supporting information


**Table S1:** Receiver operating characteristic (ROC) curve analysis of SP263 level on recurrence free (A), disease free (B) and overall survival (C) status.
**Table S2:** Univariate and multivariate analyses of recurrence‐free, disease‐free, and overall survival.
**Table S3:** Comprehensive prognostic analysis and internal validation of the PD‐L1 via bootstrapping (1000 resamples).
**Figure S1:** Distribution of programmed death ligand 1 (PD‐L1) expression assessed by SP263.

## Data Availability

The data that support the findings of this study are available from the corresponding author upon reasonable request.
