# Supplementary material for: Programmed Death‐Ligand 1 Expression Predicts Poor Prognosis in Patients With Early‐Stage Non‐Small‐Cell Lung Cancer Undergoing Stereotactic Body Radiotherapy
Source: Thorac Cancer. 2026 May 1;17(9):e70296. doi: 10.1111/1759-7714.70296 (PMC13133705; doi:10.1111/1759-7714.70296)

**Supplementary Table 1. Receiver operating characteristic (ROC) curve analysis of SP263 level on recurrence free (A), disease free (B) and overall survival (C) status.**

**(A) Recurrence free survival**

Area under the ROC curve: 0.652

| Positive if Greater Than or Equal To | Sensitivity | 1 - Specificity | Youden's Index |
| --- | --- | --- | --- |
| -1.00 | 1.000 | 1.000 | .000 |
| .50 | .533 | .308 | .226 |
| 1.50 | .533 | .205 | **.328** |
| 2.50 | .467 | .179 | .287 |
| 4.00 | .400 | .179 | .221 |
| 7.50 | .333 | .103 | .231 |
| 12.50 | .267 | .051 | .215 |
| 17.50 | .200 | .051 | .149 |
| 30.00 | .200 | .000 | .200 |
| 50.00 | .133 | .000 | .133 |
| 80.00 | .067 | .000 | .067 |
| 101.00 | .000 | .000 | .000 |

**(B) Disease free survival**

Area under the ROC curve: 0.617

| Positive if Greater Than or Equal To | Sensitivity | 1 - Specificity | Youden's Index |
| --- | --- | --- | --- |
| -1.00 | 1.000 | 1.000 | .000 |
| .50 | .467 | .250 | .217 |
| 1.50 | .400 | .167 | .**233** |
| 2.50 | .333 | .167 | .167 |
| 4.00 | .300 | .167 | .133 |
| 7.50 | .200 | .125 | .075 |
| 12.50 | .167 | .042 | .125 |
| 17.50 | .133 | .042 | .092 |
| 30.00 | .100 | .000 | .100 |
| 50.00 | .067 | .000 | .067 |
| 80.00 | .033 | .000 | .033 |
| 101.00 | .000 | .000 | .000 |

**(C) overall survival**

Area under the ROC curve: 0.653

| Positive if Greater Than or Equal To | Sensitivity | 1 - Specificity | Youden's Index |
| --- | --- | --- | --- |
| -1.00 | 1.000 | 1.000 | .000 |
| .50 | .545 | .250 | .295 |
| 1.50 | .455 | .188 | **.267** |
| 2.50 | .409 | .156 | .253 |
| 4.00 | .364 | .156 | .207 |
| 7.50 | .227 | .125 | .102 |
| 12.50 | .182 | .063 | .119 |
| 17.50 | .136 | .063 | .074 |
| 30.00 | .091 | .031 | .060 |
| 50.00 | .091 | .000 | .091 |
| 80.00 | .045 | .000 | .045 |
| 101.00 | .000 | .000 | .000 |

**Supplementary Table 2. Univariate and multivariate analyses of recurrence-free, disease-free, and overall survival**

| **Clinical factor** | **Recurrence-free survival** | | | | **Disease-free survival** | | | | **Overall survival** | | | |
| --- | --- | --- | --- | --- | --- | --- | --- | --- | --- | --- | --- | --- |
|  | **Univariable analysis** | | **Multivariable analysis** | | **Univariable analysis** | | **Multivariable analysis** | | **Univariable analysis** | | **Multivariable analysis** | |
|  | **HR (95% CI)** | ***P* value** | **HR (95% CI)** | ***P* value** | **HR (95% CI)** | ***P* value** | **HR (95% CI)** | ***P* value** | **HR (95% CI)** | ***P* value** | **HR (95% CI)** | ***P* value** |
| **Age** (years)  ≤78 vs. >78 | 0.27  (0.07–0.95) | **0.04** | 0.29  (0.08–1.08) | 0.06 | 0.58  (0.26–1.27) | 0.18 |  |  | 1.01  (0.41–2.48) | 0.98 |  |  |
| **Sex**  Male vs. female | 0.45  (0.10–2.02) | 0.30 | 0.67  (0.11–0.45) | 0.67 | 0.24  (0.06–0.99) | **0.05** | 0.69  (0.12–3.81) | 0.67 | 0.03  (0.00–2.54) | 0.12 |  |  |
| **ECOG**  0 vs. 1–2 | 1.02  (0.31–3.04) | 0.97 |  |  | 1.50  (0.69–3.25) | 0.30 |  |  | 1.77  (0.71–4.56) | 0.22 |  |  |
| **CCI**  1–0 vs. 2+ | 1.01  (0.36–2.78) | 0.99 | 0.47  (0.12–1.89) | 0.29 | 1.45  (0.68–3.12) | 0.34 | 0.67  (0.24–1.88) | 0.45 | 2.64  (0.96–7.28) | 0.06 | 1.51  (0.46–4.89) | 0.50 |
| **Smoking**  Never smoker  Former  Current | 1  0.64  (1.6–2.57)  1.28  (0.37–4.47) | 0.53  0.70 |  |  | 1  1.37  (0.48–3.93)  1.69  (0.58–4.89) | 0.55  0.33 |  |  | 1  2.66  (0.57–12.44)  3.74  (0.80–17.43) | 0.21  0.09 |  |  |
| **Histology**  Non-SCC vs. SCC | 1.67  (0.60–4.67) | 0.33 | 1.54  (0.41–5.69) | 0.52 | 2.67  (1.25–5.68) | **0.01** | 1.93  (0.80–4.65) | 0.14 | 4.13  (1.57–10.82) | **<0.01** | 3.08  (1.09–8.74) | **0.03** |
| **EGFR**  Wild type vs. mutation | 0.61  (0.17–2.17) | 0.44 |  |  | 0.28  (0.08–0.92) | **0.04** | 0.46  (0.11–1.94) | 0.29 | 0.03  (0.00–1.61) | 0.08 |  |  |
| **ROS1**  Wild type vs. mutation | 1.41  (0.44–4.50) | 0.56 |  |  | 1.68  (0.74–3.82) | 0.21 |  |  | 2.42  (0.98–5.98) | 0.06 |  |  |
| **ALK**  Wild type vs. mutation | 0.58  (0.08–4.53) | 0.61 |  |  | 0.89  (0.26–3.12) | 0.86 |  |  | 1.30  (0.35–4.91) | 0.69 |  |  |
| **Stage**  IA2 vs. IA3-IIA | 0.52  (0.19–1.45) | 0.21 |  |  | 1.26  (0.58–2.74) | 0.56 |  |  | 4.19  (1.23–14.30) | **0.02** | 3.38  (0.96–11.82) | 0.06 |
| **Response after RT**  SD vs. PR & CR | 1.36  (0.56–4.02) | 0.58 |  |  | 1.59  (0.72-3.54) | 0.25 |  |  | 2.26 (0.86–7.66) | 0.09 |  |  |
| **PD-L1**  Negative vs. positive | 2.45  (0.90–6.93) | 0.08 |  |  | 2.44  (1.14–5.21) | **0.02** |  |  | 3.03  (1.22–7.50) | **0.02** |  |  |
| <2% vs. ≥2% | 3.08  (1.11–8.55) | **0.03** | 3.34  (0.88–12.73) | 0.08 | 2.41  (1.12–5.17) | **0.02** | 2.25  (0.84–6.04) | 0.11 | 2.70  (1.11–6.60) | **0.03** | 1.36  (0.47–4.00) | 0.57 |

HR, hazard ratio; CI, confidence interval; ECOG, Eastern Cooperative Oncology Group; CCI, Charlson Comorbidity Index; SCC, squamous cell carcinoma; EGFR, epidermal growth factor receptor; ROS1, c-ros oncogene 1; ALK, anaplastic lymphoma kinase; RT, radiotherapy; SD, stable disease; PR, partial response; CR, complete response; PD-L1, programmed death-ligand 1

| **Outcome** | **PD-L1 variable** | **Univariable analysis** | | **Multivariable analysis** | | |
| --- | --- | --- | --- | --- | --- | --- |
|  |  | **HR (95% CI)** | **Bootstrap**  **P value** | **HR (95% CI)** | **Bootstrap**  **P value** | **Bootstrap BCa 95% CI for B** |
| **RFS** | Continuous | 1.06 (1.03-1.10) | <0.01 | 1.07 (1.03-1.10) | N/A | N/A |
|  | Dichotomized (2%) | 3.08 (1.11-8.55) | 0.02 | 3.34 (0.88-12.73) | 0.06 | -1.293 to 19.485 |
| **DFS** | Continuous | 1.05 (1.03-1.08) | <0.01 | 1.06 (1.03-1.09) | 0.01 | -0.128 to 0.208 |
|  | Dichotomized (2%) | 2.41 (1.12-5.17) | 0.02 | 2.25(0.84-6.04) | 0.16 | -0.472 to 2.984 |
| **OS** | Continuous | 1.04 (1.02-1.07) | <0.01 | 1.04 (1.01-1.06) | 0.01 | -0.057 to 0.130 |
|  | Dichotomized (2%) | 2.70 (1.11–6.60) | 0.04 | 1.36 (0.47-4.00) | 0.33 | -0.725 to 2.421 |

**Supplementary Table 3. Comprehensive prognostic analysis and internal validation of the PD-L1 via bootstrapping (1,000 resamples)**

HR, hazard ratio; CI, confidence interval; BCa, Bias-corrected and accelerated; RFS, recurrence free survival; DFS, disease free survival; OS, overall survival

**Supplementary Figure 1. Distribution of programmed death ligand 1 (PD-L1) expression assessed by SP263**


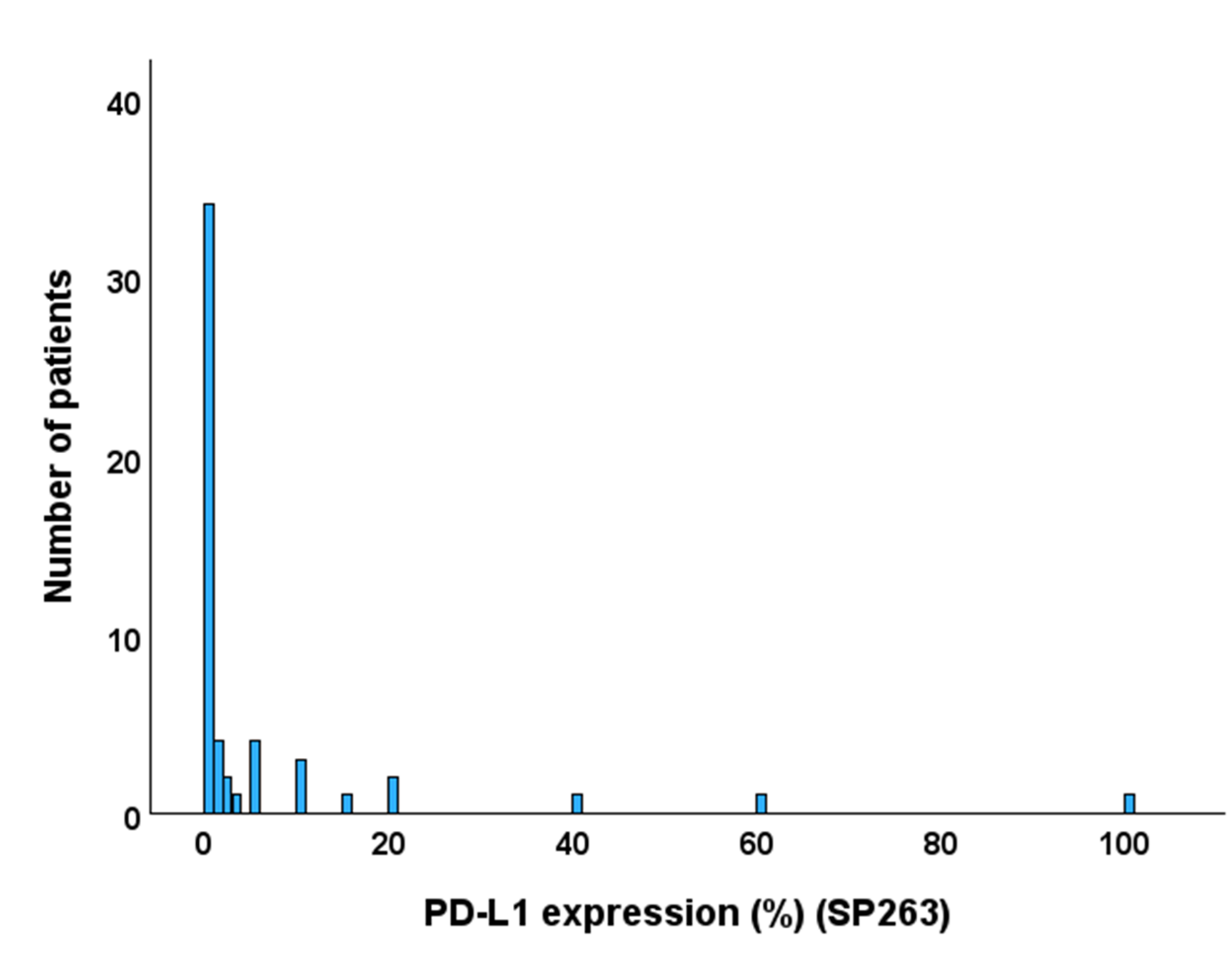

Supplement: Supplementary file 1 — Table S1: Receiver operating characteristic (ROC) curve analysis of SP263 level on recurrence free (A), disease free (B) and overall survival (C) status. Table S2: Univariate and multivariate analyses of recurrence‐free, disease‐free, and overall survival. Table S3: Comprehensive prognostic analysis and internal validation of the PD‐L1 via bootstrapping (1000 resamples). Figure S1: Distribution of programmed death ligand 1 (PD‐L1) expression assessed by SP263. [file TCA-17-e70296-s001.docx]
